# Surface Engineering of MXenes for Biomedical Uses: Functionalization Strategies and Application Trends

**DOI:** 10.34133/bmr.0327

**Published:** 2026-03-09

**Authors:** Sehyeon Park, Hee Jeong Byun, Jae Young Lee

**Affiliations:** ^1^ Department of Materials Science and Engineering, Gwangju Institute of Science and Technology, Gwangju 61005, Republic of Korea.; ^2^ GIST InnoCORE AI-Nano Convergence Institute for Early Detection of Neurodegenerative Diseases, Gwangju Institute of Science and Technology, Gwangju 61005, Republic of Korea.

## Abstract

MXenes, a class of 2-dimensional transition metal carbides and nitrides, have emerged as highly versatile materials in the biomedical field because of their high electrical conductivity, hydrophilicity, large surface-area-to-mass ratio, and compositional versatility. Despite their promise, the inherent instability in physiological environments, lack of inherent biological activity, and potential toxicity remain major challenges limiting their biomedical applications. To address these issues, a wide range of surface engineering strategies have been developed, including covalent and noncovalent functionalization with various biomolecules, biomedical polymers, and nanomaterials. Specifically, the surface modification of MXene is intended to improve biostability and biocompatibility, and confer specific biological functions for applications in tissue engineering, biosensing, antibacterial therapy, and multimodal bioimaging. This review provides a comprehensive overview of the recent advances in MXene functionalization in the biomedical field. Based on their mechanisms and biomedical functions, we categorized the functionalization strategies and proposed key design principles for the development of next-generation MXene-based therapeutic and diagnostic platforms.

## Introduction

### MXene overview

Two-dimensional (2D) nanomaterials, such as graphene, MXene, black phosphorus nanosheets, and molybdenum disulfide, have attracted marked attention for the design and production of functional biomaterials with appropriate electrical, mechanical, optical, and magnetic properties. 2D nanomaterials have large surface areas, ultrathin structures, and diverse molecular interactions [1] that render them suitable for a wide range of biomedical applications, including drug delivery, tissue engineering, and biosensing [2–5]. MXenes were first reported by Gogotsi and colleagues in 2011, and have attracted substantial interest because of their unique structural and material properties, including hydrophilicity and electrical conductivity [6,7]. MXenes are typically synthesized by selectively etching element A from the MAX phase, a family of layered ceramics with the general formula M_*n*+1_AX*_n_* (*n* = 1 to 4), where M is an early transition metal (e.g., Mo, Nb, Cr, V, and Ti), A is a group-13 or group-14 element (e.g., Al or Si), and X is carbon and/or nitrogen. The etching process is most commonly performed using hydrofluoric acid to remove the weakly bonded A layer and introduce surface termination groups (T*_x_*) (e.g., –OH, –O, and –F), resulting in the final formula M_*n*+1_X*_n_*T*_x_*. The resultant multilayered MXenes can be further delaminated into single- or few-layered nanosheets by intercalating organic molecules, such as dimethyl sulfoxide and ethanol. These MXene flakes typically exhibit enhanced dispersibility in solution and large surface areas.

MXenes possess unique electrical, structural, and interfacial properties that render them highly versatile for the fabrication of various functional materials. First, MXenes, which present atomically thin 2D layers with expanded interlayer spacing and a high specific surface area, provide a wide distribution of accessible active sites, enhancing their ability to interact with diverse ions and molecules in biological systems. Moreover, their inherent metallic conductivity and high electron mobility can be finely modulated by selecting M and X elements and/or engineering the surface termination groups [8]. For example, Mo-containing MXenes (e.g., Mo_2_TiC_2_T*_x_* and Mo_2_Ti_2_C_3_T*_x_*) exhibit semiconducting behavior (an increase in resistivity upon cooling). Surface termination is a key determinant of the electronic states of MXenes. MXene terminated with –O retains its metallic conductivity, whereas –OH termination induces the opening of a bandgap, endowing it with semiconducting characteristics [9]. On the other hand, the surface terminal groups of MXenes contribute primarily to their hydrophilicity and chemical reactivity, which further influence molecular interactions with various substances and allow for subsequent functionalization. Owing to their high surface reactivities and conductivities, MXenes are recognized as promising candidates for bioelectrodes and biosensor applications [10,11]. MXenes have also been integrated into tissue engineering scaffolds because their conductive properties, photothermal properties, reactive oxygen species (ROS)-related activity, and surface functionality are beneficial for influencing cellular behaviors, controlling drug release kinetics, and modulating biological responses for advanced tissue engineering scaffold design [12,13]. Colloidal stability, particularly in aqueous environments, is a prerequisite for solution-based processing and biomedical applications [14,15].

### Motivation of MXene functionalization

Although MXenes have shown promising characteristics for use in various biological systems, their functions in physiological environments often require further optimization for specific applications. Surface functionalization has been recognized as a key strategy for enhancing performance, including stability, biocompatibility, and functionality. For example, MXenes can be endowed with specific functionalities (e.g., therapeutic efficacy or biosensing ability) by tailoring their surfaces with biologically relevant molecules, polymers, or nanomaterials.

One of the most critical limitations of MXenes in the biomedical field is their instability, because they are susceptible to oxidation under ambient and aqueous conditions. For instance, Ti_3_C_2_T*_x_* MXenes degrade rapidly upon exposure to air or water, primarily by reacting with oxygen, leading to the formation of surface oxides (e.g., anatase-phase TiO₂). These degradation reactions typically begin at the sheet edges and progress inward. This instability significantly compromises the material characteristics and long-term stability of MXenes during processing and application [13–15]. Immobilizing or coating MXene surfaces with various stabilizing agents can effectively reduce oxidation rates, possibly prolonging MXene performance under biologically relevant conditions [16–18].

Biocompatibility is a prerequisite for biomedical applications. Nonfunctionalized pristine MXenes have been reported to be associated with adverse cellular responses, including reduced cell viability and elevated ROS levels, leading to potential damage to biological functions. Surface functionalization of MXenes with biocompatible substances (e.g., biomolecules and biomedical polymers) can passivate the toxic reactive sites on MXenes and modulate biological interactions to improve cellular responses and reduce tissue damage [19,20]. Consequently, surface engineering of MXenes can promote their safe and effective integration into biological systems.

Finally, the incorporation of new properties into MXenes can enable the production of multifunctional MXene-based biomaterials for improved performance in diverse biomedical applications, such as tissue engineering, biosensing, antibacterial therapy, bioimaging, and drug delivery. For example, the immobilization of metal nanoparticles or metal–organic frameworks (MOFs) can introduce nanozyme-like catalytic activity to mitigate excessive ROS levels. The conjugation of small biomolecules or biomedical polymers to MXenes can enable the delivery of specific biological cues for specific biological interactions (e.g., targeted drug delivery and stimuli responsiveness). Therefore, functionalization is an essential strategy to substantially expand the functional scope and application potential of MXenes.

### Scope and objectives

**Fig. 1.**
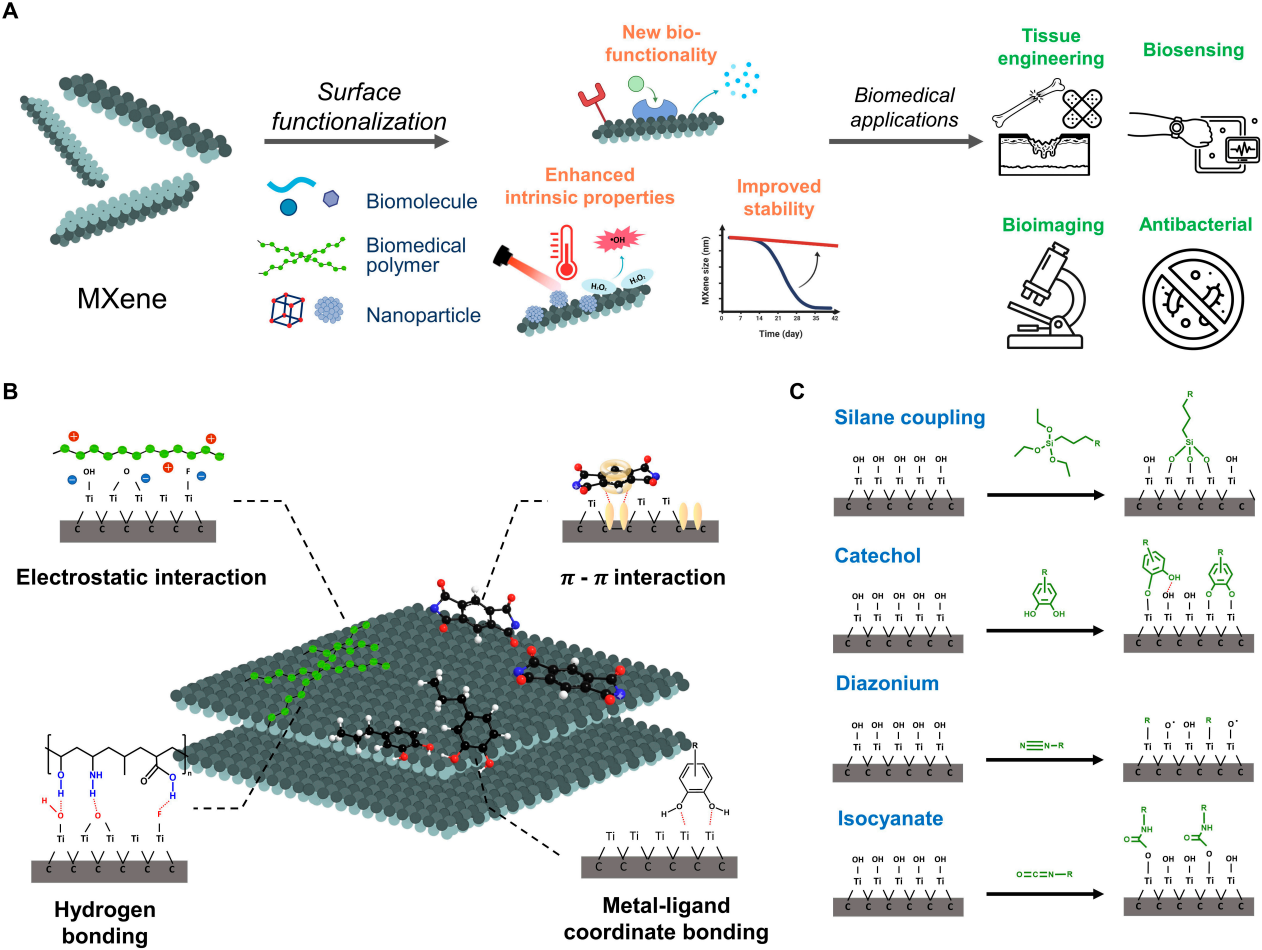
Overview of MXene surface functionalization strategies for biomedical applications. (A) Strategies, aims, and biomedical applications of MXene functionalization. (B) Noncovalent conjugation strategies for MXene functionalization. (C) Common covalent conjugation strategies for MXene functionalization.

The aim of this review is to provide a comprehensive overview of the recent advances in MXene surface functionalization, particularly in biomedical applications. We explore a wide range of modification strategies and highlight how these modifications improve the physicochemical stability, biocompatibility, and application-specific functionalities. The functionalization of MXenes is categorized depending on their specific applications, including tissue engineering, biosensing, antibacterial therapies, and bioimaging (Fig. 1A). Finally, this review provides perspectives on the next generation of MXene-based biomaterials and devices.

## MXene Functionalization: Mechanisms and Techniques

During their synthesis, MXenes naturally acquire diverse hydrophilic functional groups (e.g., –OH, –F, and –O) on their surfaces, which can be further used for both noncovalent and covalent bonding with various substances. The conjugation of biomolecules, biomedical polymers, and nanomaterials introduces new functionalities and improves the stability and biocompatibility of MXene-based materials for tissue regeneration, biosensing, imaging, and other biomedical applications.

### Conjugation

#### Noncovalent bonding strategies

The noncovalent functionalization of MXenes relies on weak intermolecular forces, such as hydrogen bonding, electrostatic interactions, π–π stacking, and metal–ligand coordinate bonding (Fig. 1B). This noncovalent functionalization approach is particularly attractive owing to the easy preparation process and preservation of the intrinsic conductivity and layered structure of MXenes [21–23].

Hydrophilic polymers [e.g., polyvinyl alcohol (PVA), polyacrylic acid (PAA), and polyethylene glycol (PEG)] are strongly adsorbed onto MXene nanosheets by forming hydrogen bonds with the surface –OH and –O groups. This modification is primarily performed to enhance colloidal and oxidation stability in aqueous media and/or provide additional functional groups for subsequent bioconjugation [24–28].

In addition, MXenes can be modified by electrostatic charge–charge interactions. The negatively charged MXene surface (zeta potential ≈ –40 mV) readily interacts with positively charged molecules [29–33]. For example, Thurakkal and Zhang [29] immobilized cationic porphyrins onto Ti_3_C_2_T*_x_* MXene through electrostatic interactions and found that functionalized MXene exhibited 5 to 17 times lower TiO_2_ formation than bare MXene, indicative of reduced MXene degradation, demonstrating substantially improved oxidation resistance. In addition to colloidal stability, the electrostatic immobilization of functional drugs and probes onto MXenes has been extensively explored for biomedical and sensing applications [30–33]. For example, positively charged drugs (e.g., doxorubicin) can be easily adsorbed onto the surfaces of MXenes and released in response to near-infrared (NIR) irradiation, enabling effective photothermal and chemodynamic therapy for tumor ablation [34–37]. Metal–ligand coordinate bonds can be formed between catechols and transition metals (e.g., Ti or Nb) in MXenes; these bonds are generally more stable than those formed by other noncovalent interactions. Polydopamine (PDA) has been widely used for metal–ligand coordination-based functionalization of MXenes [38–43]. For instance, Zheng et al. [38] coated Nb₂C MXene surfaces with PDA under mild alkaline conditions to prevent oxidation.

MXenes can also be functionalized with aromatic molecules or conjugated polymers via π–π stacking; such MXenes are being increasingly applied as batteries and catalysts [44–46]. However, this strategy has not been extensively explored for biomedical applications.

Noncovalent strategies, including hydrogen bonding, electrostatic interactions, π–π stacking, hydrophobic interactions, and metal–ligand coordination, are simple and scalable while preserving MXene’s intrinsic properties. Hence, noncovalent functionalization strategies can be used to immobilize biomolecules and improve their colloidal stability, oxidation resistance, and functional versatility. Table 1 lists several examples of noncovalent MXene functionalization for biomedical applications.

**Table 1.** Noncovalent and covalent conjugation strategies for MXene functionalization

Strategy category	Functionalization technique	MXene type	Functionalization component(s)	Application	Description	Ref.
Noncovalent	Electrostatic interaction	Ti_3_C_2_T*_x_*	Porphyrin	Oxidation resistance	Reduced MXene degradation in water	[29]
		Ti_3_C_2_	Cationic modifier (DTAB, OTAB, DDAB)	Improvement of dispersion and stability	Uniform dispersion and strong interfacial adhesion; improved thermal stability	[30]
		Ti_3_C_2_	Papain	Enzyme immobilization	Enhanced pH and thermal stability; 39.25% activity after 20 d; 50% activity after 5 reuse cycles	[33]
		Ti_3_C_2_T*_x_*	PEI	Water desalination	Surface charge induction, high salt rejection and water permeance	[153]
		Nb_2_C	PEI	Tissue engineering	Introduction of primary amine groups; formation of Schiff base formation with aldehyde groups in the hydrogel matrix	[38]
		Nb_2_C	DOX	Drug delivery	Tumor cell death with NIR-triggered hyperthermia	[36]
		Ti_3_C_2_	DOX	Drug delivery	Promoted tumor targeting and ablation	[35]
	Hydrogen bonding	Nb_2_C	PVP	Photothermal therapy	Excellent biocompatibility; degradation by myeloperoxidase	[25]
		Nb_2_C	PVP	Cancer treatment, physiological stability	Improved physiological stability biocompatibility; chemodynamic therapy	[24]
		Ti_3_C_2_T*_x_*	Ethanol, acetone, cyclohexane	Ion sieving and separation	Outstanding sub-1 nm ion rejection; selective proton–cation transport ratios	[154]
		KOH-treated Ti_3_C_2_T_*x*,_ Ti_3_C_2_T*_x_*	PVA	Mechanical property reinforcement	Extensive hydrogen bonding with O-rich MXene; improved hydrogel mechanical properties	[155]
		Ti_3_C_2_T*_x_*	PVA	Electronic skin	High stability in acidic/basic environments; high elastic modulus; pressure-sensing performance	[28]
		Ti_3_C_2_T*_x_*	DNA	Biosensing	Nucleocapsid gene detection in saliva with low detection limit and high specificity	[156]
	Metal–ligand coordinate bonding	Ti_3_C_2_T*_x_*	PDA	Solar absorption	High light absorption of solar spectrum at broad wavelength	[157]
		Ti_3_C_2_T*_x_*	PDA	Substrate adsorption and stability improvement	Strong substrate adhesion; hydrophobicity; enhanced adsorption capacity	[59]
		Ti_3_C_2_T*_x_*	ADPOA	Oxidation and stability improvement	Stable colloidal dispersions in multiple organic solvents; improved oxidation resistance	[158]
		Ti_3_C_2_T*_x_*	PDA	Mechanical and oxidative stability improvement	Tightly aligned MXene films with improved tensile strength, elongation, and conductivity; resistance to oxygen and moisture	[40]
		Nb_2_C	PDA	Tissue engineering	Hydrogel adhesion with tissue; decreased degradation	[38]
	π–π interaction	Ti_3_C_2_T*_x_*	6C-DQPZ	Energy storage	High reversibility; excellent retention	[46]
		Ti_3_C_2_	PDIsm	Photocatalysis	Facilitated π–π stacking-induced charge separation; photocatalytic oxidation	[44]
		-	PI	Energy storage	Enhanced electron transport; structural stability	[45]
Covalent	Silane coupling	Ti_3_C_2_T*_x_*	APTES, GOx, DOX, PEG	Drug delivery	Alleviation of tumor hypoxia; enhanced chemo/photothermal cancer therapy	[37]
		Ti_3_C_2_T*_x_*	APTES, lipase	Enzyme immobilization	Enhanced catalytic activity; improved pH tolerance; thermal stability; reusability	[159]
		Ti_3_C_2_	APTES, MNP	Drug delivery and tissue engineering	Multi-stimuli responsiveness; controllable drug release	[32]
		Ti_3_C_2_	APTES, Thioketal, DOX, PDA	Drug delivery	ROS- and pH-responsive drug release	[160]
		Ti_3_C_2_T*_x_*	APTES	Oxidation rate control	Improved stability in air	[161]
		Ti_3_C_2_T*_x_*	APTES	Pb^2+^ adsorption	Interaction with lead ions; increased adsorption capacity	[162]
		Ti_3_C_2_	APTES	Biosensing	Sensitive detection of carcinoembryonic antigen	[49]
		Ti_3_C_2_T*_x_*	FAS	Hydrophobicity control	Enhanced durability; self-cleaning; liquid-repellency	[163]
		Ti_3_C_2_T*_x_*	OTS	Hydrophobicity control	Colloidal stability in nonpolar solvents	[50]
		Ti_3_C_2_T*_x_*	KH570	Pb^2+^ adsorption	Increased specific surface area; thermostability; ion-exchange capacity	[164]
		Ti_3_C_2_	PFDTMS	Hydrophobicity control	Salt-blocking in MXene membrane	[165]
		Ti_3_C_2_T*_x_*	Polyelectrolyte brush, SPEEK, chitosan	Proton transfer membrane construction	Enhanced proton conductivity in membrane	[166]
	Carbodiimide coupling	V_2_C QD	PEG, TAT peptide, exosome	Bioimaging (PAI, MRI) and photothermal therapy	Induced gene/protein damage under NIR for tumor treatment; PAI and MRI	[51]
		Ti_3_C_2_T*_x_*	PEG	Stability and processability enhancement	Improved dispersibility; biocompatibility	[167]
		Ti_3_C_2_T*_x_*	Serine	Biosensing	Supramolecular hydrogen bonds for self-healing	[168]
	Catechol conjugation	Ti_3_C_2_T*_x_*	Catechol (dopamine, pyrocatechol)	Functionalization platform	Fluorescein labeling	[52]
	Isocyanate conjugation	Ti_3_C_2_T*_x_*	Dodecyl isocyanate	Hydrophobicity control	Improved nanofiller dispersion in thiourethane matrix	[169]
		Ti_3_C_2_T*_x_*	Octadecyl isocyanate	Stability enhancement	Physical stability in water	[170]
	Phosphonic acid conjugation	Ti_3_C_2_T*_x_*	Alkylphosphonic acid	Oxidation resistance control	Hydrophobicity; long-term oxidation stability; nonpolar compatibility	[171]
	Diazonium conjugation	Ti_3_C_2_T*_x_*	Diazonium	Metal ion adsorption	Ultrafast adsorption kinetics; high maximum adsorption capacities	[172]
		Ti_3_C_2_T*_x_*	Diazonium	Energy storage	Increased capacitance; reduced impedance; stable cycling	[173]

DTAB, decyltrimethylammonium bromide; OTAB, octadecyltrimethylammonium bromide; DDAB, dihexadecyldimethylammonium bromide; PEI, polyethyleneimine; DOX, doxorubicin; PVP, polyvinylpyrrolidone; PVA, polyvinyl alcohol; PDA, polydopamine; ADPOA, alkylated 3,4-dihydroxyl-l-phenylalanine; 6CN-DQPZ, diquinoxalinophenazine; PDIsm, perylene diimide supramolecular; PI, polyimide; APTES, (3-aminopropyl)triethoxysilane; GOx, glucose oxidase; PEG, poly(ethylene glycol); MNP, magnetic nanoparticle; MPTMS, (3-mercaptopropyl)trimethoxysilane; MATES, (methyl aniline) triethoxysilane; DCTES, (dodecyl) triethoxysilane; FAS, fluoroalkylsilane; OTS, octyltriethoxysilane; KH570, (3-(methacryloxy)propyltrimethoxysilane); AEAPTMS, [3-(2-aminoethylamino)-propyl]trimethoxysilane; PFDTMS, trimethoxy(1*H*,1*H*,2*H*,2*H*-per-fluorodecyl)silane; PAAm, poly(acrylamide); PMMA, poly(methyl methacrylate); PHEMA, poly(hydroxyethyl methacrylate); PDMA, poly(N,N-dimethylacrylamide); SPEEK, sulfonated poly(ether ether ketone)

Although such strategies are particularly attractive for biomedical applications, the relatively weak nature of the interactions between MXenes and adsorbed molecules often leads to eventual desorption under physiological conditions, potentially limiting long-term stability and controlled functionality [47,48]. Therefore, interaction strength and assembly conditions must be carefully designed and optimized.

#### Covalent bonding strategies

The covalent conjugation of MXenes offers more stable and specific surface modifications than noncovalent approaches. Surface termination groups, particularly hydroxyl (–OH) moieties on transition metal layers, are the primary reactive sites for covalent bonding. Among various covalent techniques, silane coupling, carbodiimide chemistry, and catechol-based immobilization have been widely employed (Fig. 1C and Table 1).

Silane functionalization is the most widely used approach. Silane derivatives, such as (3-aminopropyl) triethoxysilane (APTES) and octadecyltrichlorosilane (OTS), react with the surface hydroxyl groups on MXene to form stable M–O–Si connections. The resulting silanized MXene exhibits improved dispersibility and interfacial compatibility, and new functional groups (such as amine, thiol, and alkyl) for further MXene modification. For instance, Yang et al. [32] functionalized Ti_3_C_2_T*_x_* MXene via APTES silanization and observed a significant shift in the zeta potential from approximately –40 to +40 mV, and further used it to synthesize electrostatic complexes with negatively charged magnetic nanoparticles. Similarly, Kumar et al. [49] introduced primary amine groups to Ti_3_C_2_ MXene using APTES for the subsequent covalent conjugation of an anti-carcinoembryonic antigen. Additionally, OTS modification introduces hydrophobicity into MXene to improve its colloidal stability in organic solvents and resistance to hydrolytic degradation [50]. Overall, silane coupling improves oxidation resistance and long-term stability; however, dense silane layers may hinder subsequent biomolecule conjugation. Carbodiimide chemistry is another widely used strategy for PEGylation and peptide conjugation. The carboxyl groups on the MXene surface are activated by 1-ethyl-3-(3-dimethylaminopropyl)carbodiimide (EDC) to form O-acylisourea intermediates, which can be further stabilized by N-hydroxysuccinimide (NHS) to yield NHS esters. Subsequently, the activated ester bonds react with amines to form stable amide bonds. For example, Cao et al. [51] functionalized V₂C quantum dot (QD) MXene with 2-arm-PEG-NH₂ using EDC/NHS chemistry and further immobilized a nucleus-targeting TAT peptide onto the PEG terminus via the same coupling reaction.

Moreover, catechol and catechol derivatives have been conjugated to MXene to introduce additional functional groups (–NH₂, –COOH, and –OH) that can serve as anchoring sites for assembly with dyes, drugs, or macromolecules. Heckler et al. [52] conjugated catechols onto MXene via dehydrative condensation and used the exposed primary amines to further immobilize a fluorescent dye (fluorescein–NHS ester) onto MXene.

Covalent functionalization strategies, including silane coupling, carbodiimide chemistry, and small-molecule conjugation, provide stable modifications that improve the dispersibility, biocompatibility, and chemical versatility of MXenes. Although these approaches enhance stability and enable multifunctionality, they may also alter surface reactivity or limit further conjugation, highlighting the need for a rational design tailored to specific biomedical applications.

### Functionalization of MXene with biomedical agents

#### Conjugation with biomolecules

**Fig. 2.**
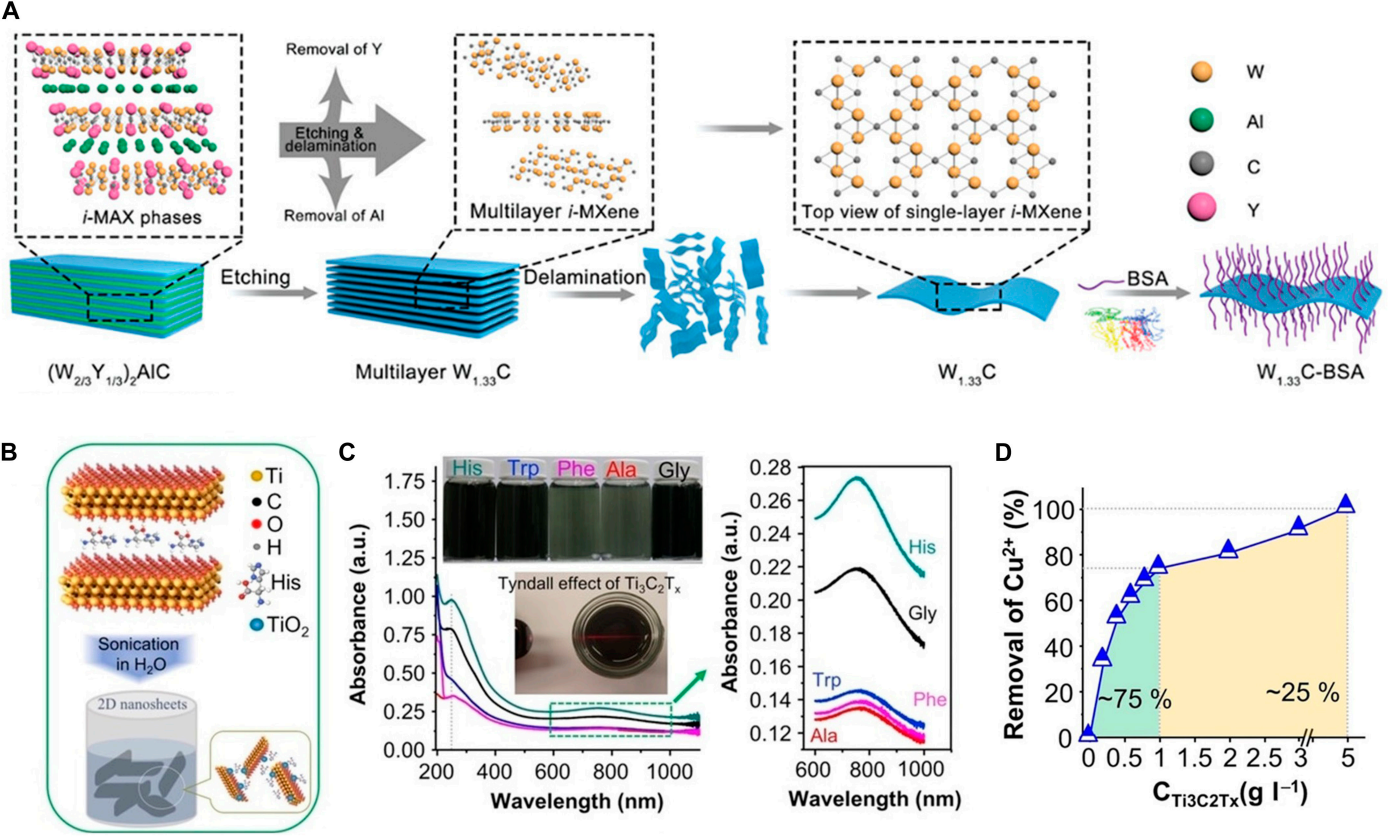
(A) Preparation of W_1.33_C–BSA nanosheets, designed as a 2D phototherapeutic platform for multimodal, imaging-guided cancer therapy. Reproduced with permission [56]. Copyright 2021, Wiley. (B) Functionalization of Ti_3_C_2_T*_x_* by amino acids, including histidine (His), tryptophan (Trp), phenylalanine (Phe), alanine (Ala), and glycine (Gly). (C) Representative photographs and ultraviolet–visible NIR spectra of amino acid-functionalized Ti_3_C_2_T*_x_* MXenes dispersed in aqueous solution. (D) Cu^2+^ removal efficiency of His-functionalized Ti_3_C_2_T*_x_*. Reproduced with permission [57]. Copyright 2020, Wiley.

Biomolecules can be immobilized on MXene surfaces using either direct covalent or noncovalent strategies. Biomolecule immobilization often restricts conformational freedom and reduces accessibility to target ligands [53–55]. This steric hindrance can compromise molecular recognition, particularly in cases where a precise spatial orientation is critical for interactions with large target biomolecules. Biomolecule-functionalized MXenes are mostly employed not for highly specific biorecognition, but for the improvement of material characteristics, such as physicochemical stability, oxidation prevention, and introduction of new functionalities (Table 2). For example, Zhou et al. [56] noncovalently conjugated bovine serum albumin (BSA) to W_1.33_C MXenes and reported improved in vivo stability, biocompatibility, and solubility (Fig. 2A). BSA-modified MXene also exhibits strong NIR absorbance, enabling multimodal tumor imaging [computed tomography (CT) and photoacoustic (PA)] and photothermal tumor ablation. Elumalai et al. [57] functionalized Ti_3_C_2_T*_x_* MXene flakes with various amino acids (histidine, tryptophan, phenylalanine, alanine, and glycine) via simple adsorption (Fig. 2B). They exhibited improved colloidal stability, regardless of the amino acid type. Interestingly, aromatic amino acids induced the formation of rutile-phase TiO₂@d-Ti_3_C_2_T*_x_* hybrids, whereas aliphatic amino acids did not induce oxidation (Fig. 2C). The histidine-functionalized TiO₂@d- Ti_3_C_2_T*_x_* hybrid could be applied for Cu^2+^ ion adsorption (Fig. 2D). Gan et al. [58] noncovalently immobilized glucose oxidase (GOx) on Ti_3_C_2_ MXene, followed by encapsulation within a poly(γ-glutamic acid)-based microneedle patch for diabetic wound healing.

**Table 2.** Functionalization of MXenes with biomolecules, biomedical polymers, and inorganic nanomaterials for diverse application

Functionalizing agent	MXene type	Functionalization component	Application	Description	Ref.
Biomolecule	Ti_3_C_2_T*_x_*	Amino acid (His, Trp, Phe, Ala, Gly)	Oxidation stability enhancement	Prevention of MXene oxidation by modification with aliphatic amino acids	[57]
	Ti_3_C_2_	ATP, Mn_3_(PO_4_)_2_	Biosensing	Uniform nanoparticle growth; fast superoxide sensing	[31]
	W_1.33_C	BSA	Bioimaging	Prolonged stability at tumor sites; enhanced in vivo CT and PA imaging	[56]
	Ti_3_C_2_T*_x_*	Gly, Leu	Inhibiting restacking	Increased interlayer spacing; improved cycling stability	[113]
	Ti_3_C_2_T*_x_*	GOx, Fe_2_O_3_	Antibacterial application	Efficient bacterial membrane disruption; promoted ROS and Fe^2+^ accumulation	[174]
	Ti_3_C_2_	GOx	Tissue engineering	Controlled GOx release to reduce glucose and pH levels	[58]
	Ti_3_C_2_	Tannic acid, Fe^2+^/Fe^3+^	Tissue engineering and antibacterial application	Catalase- and peroxidase-like activity to alleviate hypoxia	[175]
	Ti_3_C_2_	Hemoglobin	Biosensing	Facilitated Hb–NO_2_^−^ collisions; low detection limit and broad linear range	[176]
Biomedical polymer	Ti_3_C_2_T*_x_*	HA	Oxidation stability enhancement	Long-term oxidation resistance and structural stability in various solutions	[60]
	Ti_3_C_2_	HA, DOX	Drug delivery; photothermal therapy	Enhanced biocompatibility; pH-responsive drug release; tumor-specific accumulation; effective tumor ablation	[35]
	Nb_2_C	PVP	Photothermal therapy	Excellent biocompatibility; degradation by myeloperoxidase	[25]
	Ti_2_CT*_x_*	PANI, CTAB, graphene	Energy storage	Improved capacitance and cycling stability	[73]
	Ti_3_C_2_T*_x_*	PEDOT:PSS	Energy storage	Porous MXene/polymer hybrid structure; high capacitance	[177]
	Ti_3_C_2_T*_x_*	PEDOT	Energy storage	In situ polymerization of EDOT on MXene; enhanced capacitance	[178]
	Ti_3_C_2_T*_x_*	Polypyrrole	Energy storage	High volumetric capacitance; high cyclic stability	[179]
	Ti_3_C_2_	PDA	Tissue engineering; photothermal therapy	Redox homeostasis; reduced oxidative stress; bacterial elimination	[41]
	Nb_2_C	PDA	Tissue engineering; antibacterial application	Tissue adhesion; conductivity; antioxidant effects	[43]
	Ti_3_C_2_T*_x_*	PDA	Oxidation prevention	Protection MXene from water and oxygen; aqueous MXene ink stability in water for 30 d	[39]
	Ti_3_C_2_T*_x_*	PDA	Oxidation prevention, Cr (VI) removal	Effective Cr(VI) adsorption (862.3 mg/g); long-term performance	[42]
	Nb_2_C	PDA, PEI	Tissue engineering	Reduced oxidative stress; anti-inflammation; promoted myoblast maturation	[38]
	Ti_3_C_2_	PEG, GdW_10_, BSA	Photothermal therapy; bioimaging (CT, MR)	Tumor eradication without reoccurrence; dual-mode CT/MR imaging	[72]
	Ti_3_C_2_	PEG	Photothermal therapy; bioimaging (PA, CT)	Retarded degradation; PA/CT imaging; photothermal therapy	[61]
	Ti_2_C	PEG	Photothermal therapy	High photothermal efficiency; excellent biocompatibility	[62]
	Ti_3_C_2_	Soybean phospholipid, DOX	Drug delivery, photothermal therapy	High drug loading; pH-responsive and NIR-triggered on-demand drug release	[63]
	Ti_3_C_2_	Soybean phospholipid	Photothermal therapy	Photothermal ablation of tumors after IV administration	[64]
	Mo_2_C	PVA	Photothermal therapy	Fast degradability; strong NIR absorbance; photothermal efficiency	[27]
	Ti_3_C_2_T*_x_*	PVA, PDDA	Energy storage	Enhanced volumetric capacitance	[65]
	Ti_3_C_2_	PVP, DOX, DOXjade	Drug delivery; photothermal therapy	Photothermal effect; TfR down-regulation via iron chelation	[34]
	Nb_2_C	PVP, iron oxide, CaO_2_, APTES	Photothermal therapy	Production of hydroxyl radicals; photothermal-radical synergy	[24]
	Nb_2_C	PVP	Improving biocompatibility and physiological stability	Scavenging ROS; high catalytic activity against various ROS	[26]
Inorganic nanomaterial	Ti_3_C_2_T*_x_*	AuNP	Antibacterial application	Antibacterial activity for *S. aureus* and *E. coli* with a low dose	[180]
	Ti_3_C_2_T*_x_*	AuNP, GOx	Biosensing	Improved electrical conductivity; efficient electrochemical transducer	[118]
	Ti_3_C_2_	AuNP, PEG	Photothermal therapy; bioimaging (PA, CT)	Enhanced NIR absorbance (windows I and II); improved colloidal stability; radiotherapy by enhancing tumor oxygenation	[80]
	Ti_3_C_2_T*_x_*	AuNP, PEG, DOX	Photothermal therapy; drug delivery	Dual-controlled DOX release via pH response and NIR-triggered activation at tumor sites	[81]
	Ti_3_C_2_T*_x_*	Bi_2_S_3_	Antibacterial application	High antibacterial efficacy against *S. aureus* and *E. coli* under photoactivation	[85]
	Ti_3_C_2_T*_x_*	CuS, VEGF-mimicking peptide	Tissue engineering; antibacterial application	Accelerated infectious ischemic wound healing; angiogenesis	[181]
	Ti_3_C_2_	IONP, PEG, GOx	Photothermal therapy; chemodynamic therapy	Conversion of H_2_O_2_ to hydroxyl radicals; amplified photothermal and catalytic synergy for enhanced cancer treatment	[182]
	Ti_3_C_2_	IONP, soybean phospholipid	Bioimaging (MR)	High T_2_ relaxivity; high-resolution MR imaging; photothermal guidance	[183]
	Ta_4_C_3_	IONP, soybean phospholipid	Bioimaging (MR)	Superparamagnetic properties for T_2_-weighted MR imaging	[66]
	Ta_4_C_3_	MnO, soybean phospholipid	Bioimaging (MR, CT)	CT and T_2_-weighted MRI; photoacoustic imaging for tumor diagnosis	[146]
	Ti_3_C_2_	MnO*_x_*, soybean phospholipid	Bioimaging (MR)	Tumor microenvironment-responsive imaging with high contrast enhancement	[67]
	V_2_C	PtNP	Photothermal therapy; chemodynamic therapy	Strong photothermal and multi-enzyme-mimic activities; treatment of drug-resistant bacterial infections	[135]
	Nb_2_C	PtNP, DOX	Photothermal therapy; chemodynamic therapy; drug delivery	DOX release under hyperthermic and acidic conditions; inhibition of P-glycoprotein-mediated drug efflux	[36]
	Ti_3_C_2_	PtNP, PEG	Biosensing	High POD activity in the dark and under NIR irradiation	[184]
	Ti_3_C_2_T*_x_*	TiO_2_	Tissue engineering; antibacterial application	Photothermal properties; antioxidant capability; conductivity; wound dressing	[185]
	Ti_3_C_2_	VS_4_	Tissue engineering; antibacterial application	Strong chemodynamic and sonodynamic antibacterial efficacy; bone regeneration.	[186]
	Ti_3_C_2_	Fe-MOFs	Tissue engineering; antibacterial application	Promoted hydroxyl radical generation; improved chemodynamic therapy via hot electron transfer	[187]
	Ti_3_C_2_	Porphyrin-MOF	Tissue engineering; antibacterial application	ROS generation under low-intensity ultrasound; bacteria eradication; bone regeneration	[92]
	Ti_3_C_2_T*_x_*	Mn-based 1,3,5-benzenetricarboxylate-MOF	Enhancing electrical conductivity; enabling rapid ion migration	Improved bending displacement; fast response time	[188]
	Ti_3_C_2_T*_x_*	Ni_3_(HITP)_2_	Biosensing	High-fidelity EMG signal collection and electrostimulation	[91]
	Ti_3_C_2_T*_x_*	rGO	Enhancing electrical pathway	High sensitivity; fast response time; stability over 10,000 cycles; piezoresistive sensor	[100]
	Ti_3_C_2_T*_x_*	Graphene, GOx	Biosensing	Strong electrochemical catalytic capability for glucose biosensing	[99]
	Ti_3_C_2_T*_x_*	Graphene, AuNP, GOx	Biosensing	High sensitivity for glucose sensing	[189]
	Ti_3_C_2_T*_x_*	Reduced holey graphene	Biosensing	Dopamine detection with high signal retention	[119]
	Ti_3_C_2_	Quantum dot transforming, TiO_2_ opal photonic crystal, nafion	Biosensing	High sensing stability; sensitivity and selectivity for GSH detection	[190]
	Ti_3_C_2_	Quantum dot transforming	Bioimaging (fluorescence)	High fluorescence; water dispersion; stable emission; good biocompatibility	[191]
	Ti_3_C_2_T*_x_*	Quantum dot transforming	Bioimaging	Stable optical properties under varying ionic strengths and pH; photostability	[106]
	Ti_3_C_2_	Quantum dot transforming	Bioimaging	Multiplexed imaging with size-tunable emission; photostability	[192]
	Ti_3_C_2_T*_x_*	Quantum dot transforming	Chemodynamic therapy	H_2_O_2_ decomposition to hydroxyl radicals; tumor treatment	[107]
	Ti_2_N	Quantum dot transforming, soybean phospholipid	Bioimaging (PA); photothermal therapy	NIR photothermal properties with high photothermal conversion efficiency; excellent photothermal stability	[108]
	Ti_3_C_2_	Quantum dot transforming, Fe^3+^	Biosensing; bioimaging	Fluorescence nanoprobe for intracellular GSH detection	[109]
	Ti_3_C_2_	Quantum dot transforming, PLL	Biosensing; bioimaging	Fluorescence turn-off–on nanosensor for sequential detection of cyt-c and trypsin	[193]

BSA, bovine serum albumin; GOx, glucose oxidase; 4-NBD, 4-nitrobenzene-diazonium; HA, hyaluronic acid; DOX, doxorubicin; PVP, poly(vinylpolypyrrolidone); PANI, polyaniline; CTAB, cetyltrimethylammonium bromide; PEDOT, poly(3,4-ethylenedioxythiophene); PSS, poly(styrenesulfonate); PDA, polydopamine; PEI, polyethyleneamine; PEG, poly(ethyleneglycol); PVA, poly(vinyl alcohol); PDDA, poly(diallydimethylammonium chloride); APTES, (3-aminopropyl)triethoxysilane; AuNPs, gold nanoparticles; VEGF, vascular endothelial growth factor; IONP, iron oxide nanoparticle; PtNP, platinum nanoparticle; HITP, 2,3,6,7,10,11-hexaiminotriphenylene; rGO, reduced graphene oxide; PLL, poly (l-lysine)

#### Functionalization with biomedical polymers

The functionalization of MXenes with biomedical polymers is primarily aimed at preventing oxidation, improving physiological stability, enhancing biocompatibility, and introducing new functionalities. Various biomedical polymers, ranging from natural polymers [e.g., hyaluronic acid (HA)] and bioinspired polymers (e.g., PDA) to synthetic polymers [e.g., PEG, PVA, and polyvinylpyrrolidone (PVP)], have been widely employed (Table 2) [24,25,27,35,39–43,59–67].

As natural and bioinspired polymers are generally biocompatible and biologically active, MXenes modified with them will likely display biocompatible and/or bioactive characteristics [68–71]. Noncovalent functionalization of Ti_3_C_2_T*_x_* MXene with HA provides a protective barrier that restricts oxygen and water penetration and enhances the long-term antioxidation capability. Moreover, the intrinsic ROS-scavenging properties of HA contribute to the stability of MXenes [60]. Zheng et al. [38] functionalized Nb₂C MXene with PDA to suppress rapid oxidation and subsequently grafted polyethyleneimine (PEI) via electrostatic interactions. PEI-modified MXene was crosslinked with oxidized pullulan, which exhibited ROS-scavenging and immunomodulatory properties beneficial for tissue regeneration.

Functionalization of MXenes using synthetic polymers is aimed at improving colloidal stability, preventing aggregation and oxidation, enhancing biocompatibility, and providing tunable physicochemical properties for specific biomedical applications. Compared with natural polymers, synthetic polymers offer more controlled molecular structures, tunable functional groups, and superior chemical stability. Xu et al. [34] noncovalently functionalized Ti_3_C_2_ MXene with PVP to mitigate aggregation. The resulting MXene/PVP complex was further loaded with an anticancer prodrug for cancer treatment. Zong et al. [72] synthesized multifunctional Ti_3_C_2_ MXenes by immobilizing an amino-PEG derivative and subsequently conjugating BSA-coated GdW_10_ polyoxometalate clusters using EDC/NHS chemistry. The resulting nanomaterials enabled T_1_-weighted magnetic resonance (MR) and CT imaging and photothermal tumor therapy.

MXenes have also been functionalized with conductive polymers [such as polyaniline (PANI), PEDOT:PSS, and polypyrrole]. However, only a few reports have described the direct immobilization of conductive polymers on MXene surfaces in the biomedical field. Functionalization of MXenes with conductive polymers is primarily intended to increase their electrochemical capacitances. Fu et al. [73] noncovalently immobilized PANI onto Ti_2_CT*_x_* MXene via electrostatic interactions and observed significantly enhanced electrochemical capacitance. The Ti_2_CT*_x_*@PANI hybrid was further integrated with negatively charged graphene for use as an electrode material. This electrode exhibited a high specific capacitance of 635 F·g^−1^ and excellent cyclic stability of 94.25% after 10,000 cycles at 10 A·g^−1^.

Functionalization strategies using natural, synthetic, and conductive polymers stabilize MXenes, expand their biomedical utility, and enhance their electrochemical performance. These approaches have versatile applications, ranging from drug delivery and tissue regeneration to imaging and bioelectronics.

#### Functionalization with inorganic nanomaterials

The functionalization of MXenes with inorganic nanomaterials has been explored to enhance their physicochemical properties and expand their applications. Specifically, in biomedical applications, hybridization aims to (i) enhance the photothermal conversion efficiency, (ii) enable multimodal bioimaging, and (iii) introduce catalytic or enzyme-mimicking activities. The representative inorganic nanomaterials used for MXene functionalization include metallic nanoparticles, metal oxides, chalcogenides, MOFs, graphene derivatives, and QDs (Table 2).

Notably, MXene–metal nanoparticle hybrids provide strong synergistic photothermal and imaging capabilities. This enhancement is achieved because metal nanoparticles, particularly in the NIR region, absorb light through surface plasmon resonance (SPR) and convert it into heat, leading to a photothermal effect. When combined with MXenes possessing intrinsic photothermal properties, this collaborative mechanism in MXene–metal nanoparticle hybrids markedly improves the overall therapeutic efficiency [74–77]. Furthermore, conjugation with metal nanoparticles enhances the bioimaging capabilities of MXenes. The strong SPR absorption and scattering of the nanoparticles amplify the PA and optical imaging signals; nanoparticles consisting of high-atomic-number atoms increase x-ray/CT contrast [78,79]. Thus, MXene–metal nanoparticle hybrids can serve as highly effective platforms for simultaneous imaging and photothermal therapy. Tang et al. [80] synthesized Ti_3_C_2_@Au nanocomposites via the in situ reduction of HAuCl_4_ on Ti_3_C_2_ MXene and achieved enhanced absorbance in the NIR-I and NIR-II windows and strong dual-modal PA/CT contrast. Similarly, Liu et al. [81] reported the in situ growth of gold nanoparticles (AuNPs) on Ti_3_C_2_T*_x_* MXene, followed by the immobilization of doxorubicin (DOX)-linked PEG for NIR-responsive drug release and effective photothermal therapy (PTT) for tumor ablation. Tantalum carbide MXene (Ta_4_C_3_) functionalized with iron oxide nanoparticles (IONPs) demonstrated superior PTT efficiency and MR/CT imaging performance owing to synergistic magnetic contrast and Ta-based x-ray attenuation [66].

**Fig. 3.**
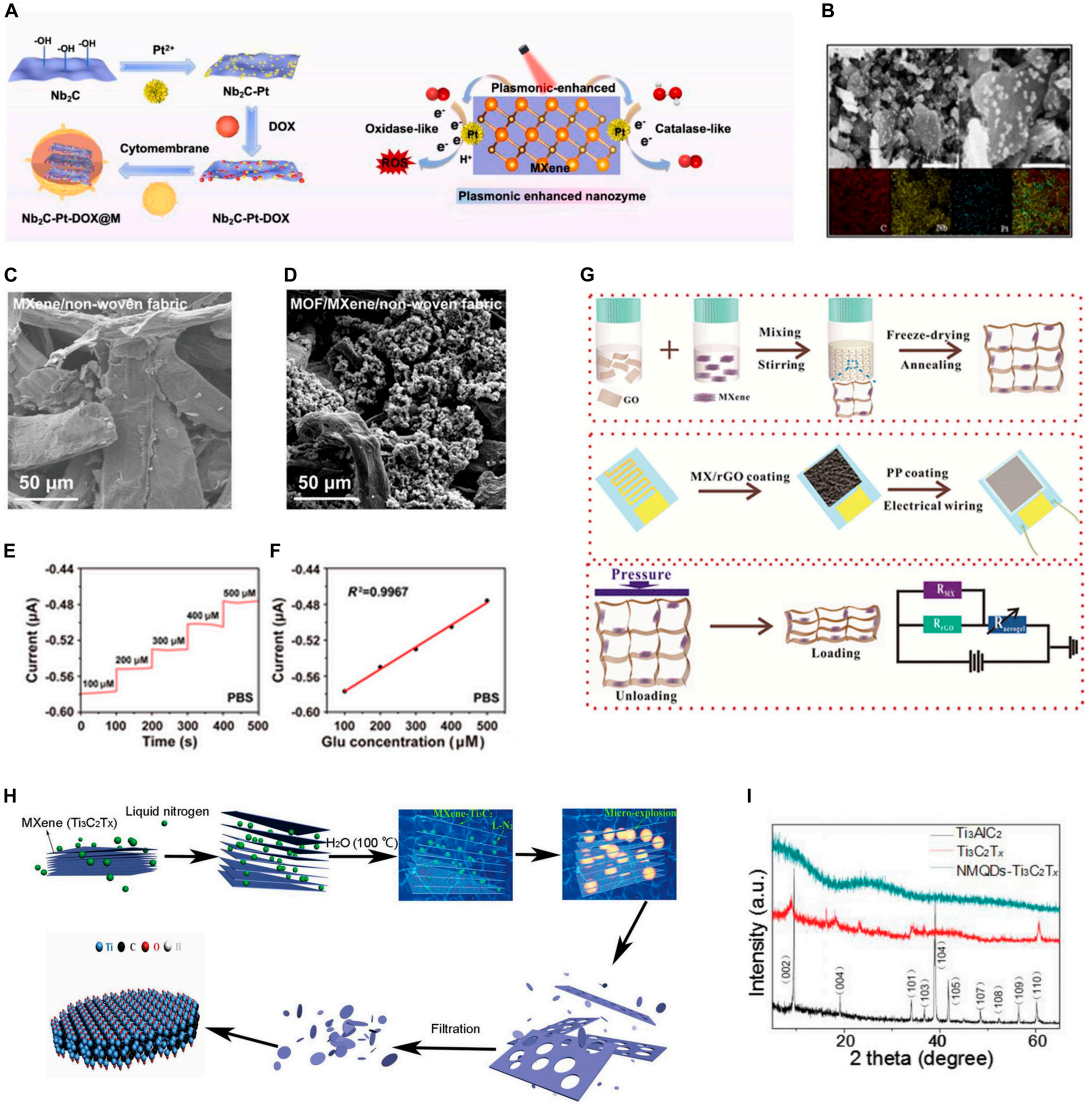
MXene functionalized with inorganic nanomaterials. (A) Biomimetic plasmonic Nb_2_C–Pt–DOX assemblies (NbPD@M) and their catalytic mechanism under NIR-II irradiation. (B) Scanning electron microscopy (SEM) images and energy-dispersive x-ray spectroscopy (EDS) element mapping of NbPD@M. Reproduced with permission [36]. Copyright 2021, Elsevier. SEM images of nonwoven fabric surfaces (C) after immersion in MXene solution and (D) following in situ anchoring of conductive MOF (Ni_3_(HITP)_2_). (E) Amperometric responses of GOx/c-MOF electrode to different glucose concentrations in 0.1 M phosphate-buffered saline (PBS). (F) Linear correlation between current changes and glucose concentrations. Reproduced with permission [91]. Copyright 2023, Wiley. (G) Schematic representation of the preparation process of the MXene/rGO aerogel (top), fabrication of the MX/rGO aerogel-based pressure sensor (middle), and corresponding piezoresistive sensing mechanism (bottom). Reproduced with permission [100]. Copyright 2018, ACS. (H) Microexplosion-mediated synthesis of nonoxidized MXene quantum dots (NMQDs-Ti_3_C_2_T*_x_*). (I) X-ray diffraction (XRD) patterns of Ti_3_AlC_2_, Ti_3_C_2_T*_x_*, and NMQDs-Ti_3_C_2_T*_x_*. Reproduced with permission [107]. Copyright 2020, Wiley.

Importantly, metal nanoparticles can facilitate enzyme-like activities, such as peroxidase- and oxidase-like reactions, because they provide active sites that catalyze chemical reactions with various substrates, while their conjugation with MXenes improves electron transfer efficiency [82–84]. Li et al. [85] developed Ti_3_C_2_T*_x_*-Bi_2_S_3_ nanorod hybrids, in which Bi_2_S_3_ generated electron–hole pairs in response to NIR irradiation and efficiently transferred the photogenerated electrons to the MXene, resulting in the production of superoxide (•O₂^−^) and hydroxyl radicals (•OH). They demonstrated the potent antibacterial performance of these nanorod hybrids with >99% eradication of *Staphylococcus aureus* and *Escherichia coli* within 10 min. Hao et al. [36] synthesized Nb_2_C-Pt-MXene nanozyme composites for pH- and temperature-responsive DOX release. These nanocomplexes combine NIR-II photothermal effects with catalase/oxidase-like activity, allowing for efficient oxygen supply, ROS generation, and chemotherapeutic efficacy (Fig. 3A and B).

MOFs are composed of metal ions coordinated to organic ligands [86]. Although their photothermal effect does not primarily arise from classical SPR as in metallic nanoparticles, certain MOFs or MOF composites exhibit efficient light-to-heat conversion. The immobilization of MOFs on MXenes can further enhance electron transfer efficiency [87]. Moreover, their porous architecture with tunable biodegradability, large surface area, and versatile cargo-loading capacity contributes to enzyme-like activities and utilities in biomedical applications [88–90]. Lin et al. [91] combined a porous conductive MOF, Ni_3_(HITP)_2_, and Ti_3_C_2_ MXene to construct a biosensing electrode capable of detecting sweat metabolites (i.e., uric acid and glucose) with high sensitivity and offering stable electromyogram recordings (Fig. 3C to F). The inherent porosity of Ni_3_(HITP)_2_ provided abundant active sites, enabling high electrochemical sensitivity and selectivity toward the target analytes, whereas the high conductivity and large electroactive surface area of both MXene and MOF reduced the skin contact impedance and enhanced the charge storage capacity. Wang et al. [92] designed a Ti_3_C_2_-Zr MOF hybrid incorporating a porphyrin sonosensitizer to generate ROS in response to ultrasound (US) stimulation for sonodynamic antibacterial therapy during osteomyelitis treatment.

Graphene is a 2D carbon nanomaterial with high electrical conductivity, large surface area, and high mechanical strength; however, it often suffers from aggregation and poor stability in physiological environments [93–95]. Graphene has been hybridized with MXenes to mitigate these drawbacks by combining the conductivity and flexibility of graphene with the hydrophilicity, surface functionality, and redox activity of MXenes. Various MXene–graphene hybrids have been developed to enhance electrochemical performance, improve enzyme/drug loading, and achieve superior sensitivity for biosensing and bioelectronic applications. The porous 3D network structures improve enzyme and drug loading efficiency, piezoresistive sensitivity, and electrochemical properties [96–98]. Gu et al. [99] fabricated a Ti_3_C_2_T*_x_* MXene–graphene hybrid glucose biosensor. Their MXene–graphene electrodes exhibited significantly improved sensitivity and reliability with stable GOx immobilization. Ma et al. [100] developed a Ti_3_C_2_T*_x_* MXene–reduced graphene oxide (rGO) composite pressure sensor that demonstrated superior piezoresistive performance compared with rGO alone (Fig. 3G).

QDs possess unique size-dependent optical and electronic properties such as strong fluorescence, high photostability, and tunable emission spectra. When MXenes are transformed into QDs, they acquire strong and stable fluorescence while retaining their intrinsic characteristics, such as conductivity, surface functionality, and photothermal properties. MXene–QDs therefore enable multimodal bioimaging, sensitive biosensing, drug delivery, and synergistic photothermal and catalytic therapies [101–105]. Zhou et al. [106] synthesized uniform Ti_3_C_2_T*_x_* MXene–QD (4 to 10 nm) with stable fluorescence (Fig. 3H and I). Li et al. [107] synthesized nonoxidized MXene–QDs that exhibited potent catalytic activity to convert hydrogen peroxide (H₂O₂) to toxic hydroxyl radicals (•OH) and suppress tumor growth in vitro. Shao et al. [108] synthesized Ti₂N MXene–QDs (approximately 5 nm) coated with soybean phospholipids for PA imaging-guided PTT under NIR-I/II irradiation. Luo et al. [109] synthesized primary amine-rich Ti_3_C_2_ MXene–QDs via a hydrothermal reaction in the presence of ethylenediamine. These hybrids could coordinate Fe^3+^ ions and exhibit glutathione (GSH)-responsive fluorescence recovery as a selective biosensor.

Functionalization of inorganic nanomaterials, including metallic nanoparticles, MOFs, graphene, and QDs, provides MXenes with enhanced imaging, therapeutic, sensing, and catalytic capabilities. By leveraging the synergistic interactions between MXenes and inorganic domains, these hybrids enable multifunctional biomedical platforms with promising applications in cancer therapy, antibacterial treatment, biosensing, and bioelectronics.

## Applications of Functionalized MXene

### Tissue engineering scaffolds and regenerative medicine

The unique characteristics of MXenes, such as their electrical conductivity, diverse molecular interactions, and intrinsic ROS-scavenging capabilities, make them attractive materials in tissue engineering and regenerative medicine. Their electrical properties are particularly beneficial for electroactive tissues such as nerves and muscles, in which electrical cues play critical roles in the proliferation, differentiation, and functional maturation of various types of cells. Pristine or minimally processed MXene nanosheets have been widely used in various composite scaffolds (e.g., hydrogels and fibers) [110,111]. Although most studies to date have focused on pristine MXenes, functionalized MXenes modified with nanoparticles, biomedical polymers, or biomolecules remain relatively underexplored in the field of tissue engineering. Most current applications in tissue regeneration have focused on wound healing and bone regeneration, with relatively few studies conducted on other tissue types (Table 3).

**Table 3.** Functionalization strategies for MXenes in biomedical, biosensors/wearable bioelectronics, antibacterial activity, and bioimaging applications

Application area	Target tissue/analyte	MXene type	Functionalization method	Scaffold/carrier/device component(s)	Key function/outcomes	Ref.
Tissue engineering and regenerative medicine	Bone (in vitro)	Ti_3_C_2_T*_x_*	Covalent silane coupling via GOPS	PEDOT:PSS	Combined treatment with electrical stimulation; enhanced expression of osteogenic-specific genes; promoted osteogenic differentiation	[194]
		Ti_3_C_2_T*_x_*	Noncovalent hydroxyapatite	Ti-6Al-4V	Porous hydroxyapatite structure; corrosion resistance; promoted cell-spreading	[195]
		Ti_3_C_2_T*_x_*	Noncovalent sorafenib	Silk fibroin methacrylate	NIR-induced heating and sorafenib release; bone cancer cell treatment; enhanced attachment and proliferation of osteoblasts	[196]
		Ti_3_C_2_T*_x_*	QD transformation and noncovalent DOX	Hydroxyapatite	MXene–QDs loaded with DOX on hydroxyapatite hollow microspheres; strong fluorescence; dual responsiveness to pH and NIR	[197]
	Bone	Ti_3_C_2_	Noncovalent metal NP (VS_4_)	None	Peroxidase-like activity; antibacterial activity; bone regeneration	[186]
		Ti_3_C_2_	Noncovalent porphyrin-based MOF	None	Porphyrin-based MOF; ROS generation in response to low-intensity ultrasound; antibacterial effects; promoted osteogenic differentiation	[92]
	Skeletal muscle	Ti_3_C_2_T*_x_*	Noncovalent metal NP (MnO_4_)	Pluronic F127	Conversion of ROS to O_2_; regulation of macrophage polarization toward the M2 phenotype; promoted myoblast proliferation and differentiation	[112]
		Nb_2_C	Noncovalent polymer (PDA, PEI)	Oxidized-pullulan	Electroactive Nb_2_C-based hydrogel; enhanced myoblast and MSC neural differentiation; ROS scavenging; muscle tissue repair	[38]
	Wound healing	Ti_3_C_2_	Noncovalent tannic acid and Fe^2+^/Fe^3+^	Quaternary ammonium methacryloyl acylated chitosan	Peroxidase-like activity; catalase-like activity for oxygen generation; alleviation of hypoxia; promoted healing	[175]
		Ti_3_C_2_	Covalent silane coupling via APTES and noncovalent MnO_2_	PNIPAM, alginate	PTT under NIR or AMF; AgNP release for localized antibacterial therapy	[32]
		Ti_3_C_2_	Noncovalent GOx	PGA	Reduction of local glucose levels and pH; delivery into deep tissues using a microneedle patch; reduction of local ROS levels; effective wound healing	[58]
		Ti_3_C_2_	Noncovalent PDA and HbO_2_	Hyaluronic acid-graft-dopamine	ROS scavenging; antibacterial properties; M2 macrophage polarization; oxygen release upon photothermal stimulation	[41]
		Nb_2_C	Noncovalent PDA	Pluronic F127 aldehyde, branched poly glycerine	Antioxidant activity; reduction of oxidative stress; MRSA-infected wound healing	[43]
Biosensors and wearable bioelectronics	Superoxide	Ti_3_C_2_	Noncovalent small molecules (ATP and Mn_3_(PO_4_)_2_)	GCE	Rapid detection of superoxide secreted by HepG2 cells	[31]
	Glucose	Ti_3_C_2_T*_x_*	Noncovalent AuNP and GOx	GCE	Enhanced electron exchange; linear detection of glucose; high sensitivity	[118]
		Ti_3_C_2_T*_x_*	Noncovalent graphene and GOx	GCE	Tunable porosity; GOx immobilization; enhanced electrochemical activity to glucose	[99]
		Ti_3_C_2_T*_x_*	Noncovalent graphene, AuNP, and GOx	GCE	Porous film construction for GOx immobilization; enhanced electron transmission and reduced redox potential; high-sensitivity glucose detection	[189]
	Uric acid, glucose	Ti_3_C_2_T*_x_*	Noncovalent Ni_3_(HITP)_2_ MOF	Nonwoven fabric	Low skin contact impedance; high charge storage capacity; high SNR detection of glucose and uric acid signals from sweat; effective electrostimulation for muscle theranostics	[91]
	Nitrite	Ti_3_C_2_	Noncovalent hemoglobin	GCE	Immobilization of hemoglobin onto MXene; mediator-free biosensor for nitrite detection	[176]
	Dopamine	Ti_3_C_2_T*_x_*	Noncovalent reduced holey graphene	GCE	Formation of MXene and holey reduced graphene composite; enhanced hydrophilicity; prevention of nonspecific adsorption; facilitated mass transport; dopamine detection	[119]
	Pressure	Ti_3_C_2_T*_x_*	Noncovalent rGO	PI interdigital electrode	Incorporation of MXene into rGO; 3D porous structure; enhanced piezoresistive performance; high sensitivity; fast response time; cycling stability	[100]
		Ti_3_C_2_	Noncovalent rGO	PET	rGO and MXene coating onto PET; low resistance; high sensitivity; excellent pressure linearity; fast response time; short recovery time	[198]
Antibacterial activity	PTT, drug delivery	Ti_3_C_2_	Noncovalent tannic acid and AgNP	None	AgNP formation on MXene via tannic acid-based MPN; NIR-triggered and pH-responsive AgNP release; antibacterial and anti-inflammatory effects	[134]
	PTT	Ti_3_C_2_	Noncovalent AuNP	None	High photothermal conversion efficiency	[199]
		Ti_3_C_2_T*_x_*	Noncovalent AuNP	None	Vis-NIR light-induced antibacterial effects	[180]
	PTT, PDT	Ti_3_C_2_T*_x_*	Noncovalent indocyanine green	None	Antibacterial effects; synergistic photothermal effect and ROS generation	[200]
		Ti_3_C_2_T*_x_*	Noncovalent Bi_2_S_3_	None	Accelerated charge transfer under NIR irradiation; enhanced generation of ROS; antibacterial activity; photothermal effect under NIR	[85]
	PTT, CDT	Ti_3_C_2_	Noncovalent Fe-based MOF	None	Photothermal heating; hot electron generation; promoted the Fenton reaction and hydroxyl radical generation; antibacterial activity; enhanced wound healing	[187]
		V_2_C	Noncovalent PtNP	None	High photothermal conversion efficiency; oxidase- and peroxidase-like activity under NIR-II irradiation; promoted hydroxyl radical generation; antibacterial effect	[135]
	PTT, PDT, CDT	Ti_3_C_2_	Noncovalent Fe_2_O_3_ and GOx	None	NIR-induced photothermal therapy, ROS generation, and Fe^2+^ release; consumption of glucose to activate AMPK in macrophages	[174]
Bioimaging	T_2_-weighted MR imaging	Ti_3_C_2_	Noncovalent IONP and soybean phospholipid	None	High T_2_ relaxivity; high photothermal conversion efficiency; MR imaging; photothermal cancer therapy	[183]
		Ta_4_C_3_	Noncovalent IONP and soybean phospholipid	None	Strong T_2_-weighted MR imaging contrast; high photothermal conversion efficiency; tumor ablation	[66]
	T_1_-weighted MR, CT, and photoacoustic imaging	Ta_4_C_3_	Noncovalent MnO*_x_* and soybean phospholipid	None	Ta-mediated CT imaging; superior T_2_-weighted MR imaging; photothermal property-driven photoacoustic imaging; photothermal cancer therapy	[146]
	T_1_-weighted MR and photoacoustic imaging	Ti_3_C_2_	Noncovalent MnO*_x_* and soybean phospholipid	None	pH-responsive T_1_-weighted MRI in the mildly acidic tumor microenvironment; photoacoustic imaging; photothermal tumor ablation	[67]
	T_1_-weighted MR and CT imaging	Ti_3_C_2_	Noncovalent PEG and GdW_10_; covalent BSA	None	Primary amine-presenting Ti_3_C_2_ MXene; functionalization with BSA-encapsulated GdW_2__0_; dual-modal MR/CT imaging	[72]
	Fluorescence imaging	Ti_3_C_2_	Quantum dot transforming	None	Ti_3_C_2_ quantum dots with a width of 2–5 nm and thickness of 0.5–1.5 nm; excitation in the 340–500-nm range and emission at 460–580 nm; fluorescent imaging agents	[191]
		Ti_3_C_2_T*_x_*	Quantum dot transforming	None	Synthesis of Ti_3_C_2_T*_x_* QD via a solvothermal method; highly stable fluorescence emission under varying ionic strength and pH conditions	[106]

GOPS, 3-glycidoxypropyltrimethoxysilane; PEDOT, poly(3,4-ethylenedioxythiophene); PSS, poly(styrenesulfonate); DOX, doxorubicin; PDA, polydopamine; PEI, polyethyleneimine; APTES, (3-aminopropyl)triethoxysilane; PNIPAM, poly(N-isopropylacrylamide); GOx, glucose oxidase; PGA, polyglycolic acid; PDA, polydopamine; MRSA, methicillin-resistant *S. aureus*; GCE, glassy carbon electrode; rGO, reduced graphene oxide; HITP, 2,3,6,7,10,11-hexaiminotriphenylene; PI, polyimide; PET, polyester filament; PTT, photothermal therapy; PDT, photodynamic therapy; CDT, photochemotherapy; AgNP, silver nanoparticle; MPN, metal-polyphenol network; IONP, iron oxide nanoparticles; BSA, bovine serum albumin

In addition to their conductive properties, functionalized MXenes exhibit photothermal and catalytic properties that enable stimuli-responsive therapeutic applications. Their photothermal activity has been exploited for light-triggered drug release and the photothermal ablation of bacteria and cancer cells. On the other hand, hybridization with inorganic nanoparticles introduces enzyme-mimicking catalytic activity to facilitate tissue regeneration. Yang et al. [32] developed a stimuli-responsive antibacterial hydrogel. They covalently conjugated APTES to Ti_3_C_2_ MXene and immobilized MnO₂ nanoparticles (MNPs@MXene) via electrostatic interactions and embedded MNPs@MXene and silver nanoparticles (AgNPs) in a thermo-responsive poly(N-isopropylacrylamide)/alginate hydrogel. Under NIR irradiation or an alternating magnetic field, localized heating of MNPs@MXene induced hydrogel shrinkage and triggered AgNP release on demand at diabetic wound sites. Zheng et al. [112] modified Ti_3_C_2_T*_x_* MXene through in situ MnO_2_ formation, followed by poly-l-lysine coating and crosslinking with aldehyde-functionalized Pluronic F127. The hydrogel scaffold effectively scavenged excessive ROS, promoted macrophage polarization toward an anti-inflammatory phenotype, and enhanced myoblast proliferation and differentiation. Wang et al*.* [92] reported Ti_3_C_2_ MXene functionalized with a porphyrin-based MOF for bone regeneration. The MOF@MXene hybrid generated ROS and sonocurrents upon US stimulation and accelerated osteogenesis at infected bone defect sites.

The surface engineering strategies employed in these tissue engineering applications offer distinct advantages but also present inherent limitations. Covalent and noncovalent functionalization using biomedical polymers markedly improves the interfacial compatibility between the MXenes and biological tissues. However, a primary challenge is the potential reduction in the intrinsic electrical conductivity of MXenes when they are extensively encapsulated by insulating organic layers, which may diminish the efficacy of electrical cues for electroactive cells [113,114]. The stability of functionalized MXenes within the physiological environment is a critical consideration for the design of tissue engineering scaffolds. While functionalization strategies often serve the purpose of providing a protective barrier against rapid hydrolytic oxidation, maintaining the scaffold’s functional properties (e.g., conductivity and mechanical strength) throughout the entire tissue remodeling process remains challenging [115]. The metabolic fate of the degradation products also remains a concern. The accumulation of transition metal ions (e.g., Ti and V) during scaffold resorption necessitates dose optimization to avoid localized toxicity [116,117]. Notably, as most degradation studies have focused on Ti-containing MXenes, in-depth studies with other MXene compositions will be necessary to clearly understand the degradation mechanisms, kinetics, and toxicities.

### Biosensing and bioelectronic applications

MXene-based biosensors have been developed to detect clinically relevant biomarkers, including glucose (diabetes management), superoxide (oxidative stress evaluation), and nitrite (inflammation monitoring). Effective biosensing requires high reactivity toward target analytes and efficient signal transduction. To achieve selective and sensitive detection, MXene surfaces are frequently modified with enzymes (e.g., GOx) or catalytically active nanomaterials for analyte-specific recognition and signal generation (Table 3). For example, Rakhi et al. [118] noncovalently immobilized GOx onto Ti_3_C_2_T*_x_* MXene and further formed AuNPs to enhance the electron transfer efficiency. The resulting MXene-based glassy carbon electrode demonstrated a wide linear detection range (0.1 to 18 mM), high sensitivity (4.2 μA·mM^−1^·cm^−2^), and low detection limit (5.9 μM) of glucose. Zhang et al. [119] developed an electrode composed of Ti_3_C_2_T*_x_* MXene and reduced holey graphene for dopamine detection. Pristine MXene exhibits a low sensing capability owing to its low stability and insufficient number of active sites. In contrast, MXene combined with reduced holey graphene provided a porous, interconnected network that supported electrostatic interactions and mass transport. This composite electrode successfully detected dopamine over the range of 0.2 to 125 μM, with a low detection limit of 0.044 μM.

In addition to biochemical sensing, MXenes have been employed in bioelectronic devices such as motion-sensing pressure and strain sensors. MXene/graphene-based composites are particularly attractive because their 3D porous architectures can enhance piezoresistive properties [120–122]. Ma et al. [100] reported a hybrid MXene/rGO aerogel prepared by freeze-drying and annealing. The composite material exhibited a rich porous structure and excellent piezoresistive behavior. The sensor exhibited high sensitivity (22.56 kPa^−1^), rapid response (<200 ms), and long-term durability over 10,000 cycles.

A primary limitation in biosensing is interfacial fouling, in which nonspecific protein adsorption can mask active sites and reduce sensitivity over time [123,124]. In practical bioelectronic applications, sensors may suffer from signal hysteresis and baseline drift caused by mechanical fatigue and fluctuating contact resistance between conductive components, including MXene sheets [125]. Maintaining long-term electrochemical stability remains a critical hurdle, as MXene nanosheets are highly susceptible to oxidative degradation when exposed to physiological environments or sweat. Such oxidation shifts the baseline resistance and compromises the reliability of diagnostic data [126]. While MXene flakes are generally considered biocompatible, the leaching of fragments into the skin or circulatory system poses a potential risk, as the released fragments could trigger localized inflammation or oxidative stress in surrounding tissues. Therefore, ensuring strong interfacial bonding within the hybrid complex is essential to prevent material shedding and to maintain the functional fidelity of the bioelectronic device [117,127].

### Antibacterial applications

MXenes exhibit intrinsic antibacterial activity originating from their photothermal, photodynamic, and structural properties. Their hydrophilic surfaces promote bacterial adhesion, whereas their sharp nanosheet edges physically disrupt bacterial membranes, leading to membrane damage and cell lysis [128,129]. In addition, under NIR irradiation, MXenes generate localized heat that causes bacterial membrane rupture [130–132]. Photoexcited electrons and holes on MXene surfaces can react with oxygen or water to produce ROS, which in turn oxidize bacterial membranes and cell walls. However, due to their narrow bandgap and rapid electron–hole recombination, pristine MXenes often show limited ROS generation and modest antibacterial efficacy [133]. To address these issues, MXenes have been functionalized with metal nanoparticles or MOFs for enhancing the photothermal conversion efficiency or introducing enzyme-like catalytic activity (Table 3). For example, Zhou et al. [134] constructed a tannic acid–Fe^3+^ metal–polyphenol network (MPN) on MXene, followed by the in situ formation of AgNP. This hybrid exhibited synergistic antibacterial effects through NIR-triggered AgNP release and pH-responsive MPN degradation, resulting in enhanced Ag^+^ delivery and effective bacterial eradication. Similarly, He et al. [135] immobilized Pt nanoparticles (PtNPs) on V_2_C MXene via in situ synthesis. The MXene@PtNP composite showed a markedly enhanced photothermal conversion efficiency (from 29.4% to 59.6%) and oxidase- and peroxidase-like catalytic activities under NIR-II irradiation. This catalytic–thermal synergy enabled the effective elimination of methicillin-resistant *S. aureus* and promoted tissue regeneration in infected corneal injury models.

Notably, the control of photoexcited carriers involves a fundamental technical trade-off. For example, while the rapid electron-hole recombination inherent in MXenes is highly beneficial for generating heat, it substantially hinders the generation of radicals that require carrier separation [85]. Achieving an optimal balance between these 2 competing pathways remains a challenge. Additionally, photochemotherapy (CDT)-based strategies often require specific microenvironmental conditions (e.g., acidic pH or presence of H_2_O_2_), which may limit their efficacy in neutral physiological conditions [136,137]. A major consideration in this application is the photostability of the MXene substrate. Repeated or high-intensity NIR irradiation can accelerate the oxidation of MXene nanosheets into TiO_2_, which eventually diminishes their photothermal potency and catalytic activity over multiple cycles [138]. In addition, while ROS are potent antibacterial agents, excessive production can cause collateral damage to surrounding cells [132,139]. Therefore, developing on-demand or targeted strategies is highly desired. Leaching of metal ions or secondary nanomaterials can lead to localized/systemic toxicity [140]. Hence, ensuring that the therapeutic agent remains localized to the infection site is a prerequisite for the safe clinical transition of these antibacterial platforms.

### Bioimaging platforms

Functionalized MXenes have been increasingly explored as bioimaging agents, with strategies broadly classified into 2 categories: (a) surface modification to enhance contrast in CT and MR imaging (MRI) and (b) transformation into QDs for optical imaging (Table 3).

In CT imaging, contrast enhancement is associated with the x-ray attenuation capability, which increases with the atomic number (Z) of the material component [141–143]. Hence, the functionalization of MXenes with high-Z elements (e.g., gold or bismuth) markedly enhances x-ray absorption and CT contrast. In MR, pristine MXenes typically display limited magnetic behavior with weak contrast. Magnetic nanoparticles such as manganese dioxide (MnO_2_) and iron oxide (Fe_3_O_4_) have been immobilized onto MXene surfaces to improve the T_1_- or T_2_-weighted contrast through the enhanced relaxation of surrounding water protons [144,145]. For example, Dai et al. [146] reported a multifunctional imaging and therapeutic platform based on Ta_4_C_3_ MXenes (Z = 73), which offered stronger x-ray attenuation than Ti-based MXenes (Z = 22). MnO*_x_* nanoparticles were immobilized in situ on the MXene, and the surface was further functionalized with soybean phospholipids to improve colloidal stability. In 4T1 tumor-bearing mice, the composite exhibited robust CT contrast due to Ta, effective T_1_-weighted MR enhancement from paramagnetic MnO*_x_*, and PA imaging capability.

MXenes can be engineered into QDs (MQDs) (typically < 10 nm), in which quantum confinement induces discrete energy levels. Fluorescence emission occurs through electron transitions between quantized states, and the emission wavelength can be tuned by controlling their size [147]. Compared with conventional organic fluorophores, MQDs offer superior photostability, tunable emission, and structural robustness; thus, they are attractive for long-term high-resolution imaging [148,149]. Zhou et al. [106] synthesized Ti_3_C_2_T*_x_*-derived MQDs using a solvothermal method. The MQDs exhibited strong and stable fluorescence in aqueous solutions, glycol, and PVP. Notably, unlike conventional dyes, MQDs maintained their fluorescence intensity with minimal quenching, highlighting their promise as durable fluorescent probes for bioimaging.

Despite these benefits, a major technical hurdle is the trade-off between contrast ability and colloidal stability. Most functionalized MXenes with contrast agents rely on soybean phospholipid conjugation to ensure stability; however, excessive loading of contrast agents can exceed the stabilization capacity of the phospholipid layer [146,150]. Furthermore, noncovalent conjugation of phospholipid may lead to eventual loss of interfacial bonding strength [21]. The most primary concern in biosafety is the long-term accumulation of nonbiodegradable high-Z elements (e.g., Ta and Bi) within the reticuloendothelial system (RES) particularly the liver and spleen. These heavy inorganic elements lack efficient renal clearance pathways [151,152]. Therefore, long-term metabolic fate and potential chronic toxicity of these accumulated metals must be carefully evaluated to resolve the primary hurdles for clinical approval.

## Conclusion and Perspective

Over the past decade, MXenes have emerged as a highly promising class of 2D nanomaterials with broad applications in biomedical engineering. Their intrinsic properties, including high conductivity, surface functionality, hydrophilicity, and tunable reactivity, make them attractive building blocks for applications in tissue engineering, drug delivery, antibacterial therapy, biosensing, and bioimaging. Surface functionalization strategies have enabled the innovative design of stable and multifunctional MXene-based platforms capable of performing complex biological functions.

To fabricate functional MXene-based biomaterials for specific biomedical requirements, surface engineering should be guided by overarching principles that balance material stability, biocompatibility, and biological functionality.

Tissue engineering: The primary objective is the creation of a bioactive interface through the functionalization of biomolecules, biomedical polymers, or other components to actively promote cellular interactions and tissue regeneration. So far, most functionalized MXenes have been focused on bone and wound regeneration; hence, their efficacy in other biological tissue environments needs to be further explored.

Biosensing and wearable bioelectronics: MXene functionalization primarily focuses on selective signal transduction by enhancing electron exchange pathways and reducing interfacial impedance through the functionalization of specific recognition elements, such as enzymes and/or conductive nanomaterials. Despite promising improvement in sensitivity, current strategies are still insufficient for ensuring long-term signal stability and preventing interference from nonspecific protein adsorption, which can compromise detection fidelity over extended periods.

Antibacterial applications: Synergistic therapeutic enhancement can be achieved by hybridizing MXenes with metal-based nanoparticles or MOFs by increasing the efficiency of PTT, photodynamic therapy (PDT), or CDT. Nevertheless, a strategic balance between carrier recombination and separation pathways is essential. While the rapid recombination in MXenes facilitates efficient photothermal conversion, it limits the ROS quantum yield for PDT and CDT. Thus, sophisticated interfacial engineering is essential to tune the electronic state for optimal therapeutic synergy.

Bioimaging platforms: Contrast and optical versatility could be amplified by incorporating high-atomic-number elements for CT/MR or transforming MXenes into QDs for fluorescence imaging. However, a persistent hurdle involves the lack of systematic evaluation regarding the NIR-II absorbance and imaging efficiency of diverse MXene compositions beyond Ti_3_C_2_ (e.g., Hf_2_C, V_4_C_3_, Cs_2_C, and Ti_4_N_3_), which is essential for deep-tissue diagnostics.

To address the aforementioned multifaceted limitations and bridge the gap toward clinical translation, a more structured framework for future development is imperative. By integrating materials science with systematic safety evaluations and scalable design, the following perspectives provide key guidelines to steer research toward next-generation MXene-based biomedical technologies

1. Establishing long-term clinical safety and biocompatibility: Systematic, long-term investigations in large-animal models are essential to establish the safety profile required for clinical adoption.
2. Advancing deep-tissue theranostics via NIR-II expansion: The NIR-II biowindow absorbance and imaging efficiency of diverse MXene compositions beyond Ti_3_C_2_ must be clearly evaluated.
3. Transitioning to precision chemical engineering: The development of more robust covalent conjugation techniques is necessary to ensure the structural stability and functional precision of MXene-based applications.
4. Standardization of scalable and high-purity manufacturing: Strategic progress in scalable synthesis and high-purity processing is fundamental for industry-scale production and regulatory approval.

In conclusion, MXenes are powerful and versatile material platforms with the potential to transform biomedical technologies. Their future success will depend on addressing current limitations through interdisciplinary collaboration across materials science, biology, and engineering. With strategic progress in functionalization, safety evaluation, and platform design, functionalized MXenes are poised to play a key role in next-generation biomedical devices and therapies.

## Data Availability

Data will be made available on request.
